# Process for Quality Management of Electronic Medical Records–Based Data: Case Study Using Real Colorectal Cancer Data

**DOI:** 10.2196/73884

**Published:** 2025-11-13

**Authors:** NaYoung Park, Kyungmin Na, Woongsang Sunwoo, Jeong-Heum Baek, Youngho Lee, Suehyun Lee, Hyekyung Woo

**Affiliations:** 1 Department of Health Administration, Kongju National University, Gongju-Si, Chungcheongnam-do, Gongju, 32588, Republic of Korea, 82 41-850-0328; 2 Office of eHealth Research and Business, Seoul National University Bundang Hospital, Seongnam-si, Republic of Korea; 3 Department of Computer Engineering, College of IT Convergence, Gachon University, Seongnam, Republic of Korea; 4 Department of Otorhinolaryngology, Gil Medical Center, Gachon University, College of Medicine, Incheon, Republic of Korea; 5 Division of Colon and Rectal Surgery, Department of Surgery, Gil Medical Center, Gachon University, College of Medicine, Incheon, Republic of Korea

**Keywords:** quality management, medical data, real-world data, colorectal cancer, data quality

## Abstract

**Background:**

As data-driven medical research advances, vast amounts of medical data are being collected, giving researchers access to important information. However, issues such as heterogeneity, complexity, and incompleteness of datasets limit their practical use. Errors and missing data negatively affect artificial intelligence–based predictive models, undermining the reliability of clinical decision-making. Thus, it is important to develop a quality management process (QMP) for clinical data.

**Objective:**

This study aimed to develop a rules-based QMP to address errors and impute missing values in real-world data, establishing high-quality data for clinical research.

**Methods:**

We used clinical data from 6491 patients with colorectal cancer (CRC) collected at Gachon University Gil Medical Center between 2010 and 2022, leveraging the clinical library established within the Korea Clinical Data Use Network for Research Excellence. First, we conducted a literature review on the prognostic prediction of CRC to assess whether the data met our research purposes, comparing selected variables with real-world data. A labeling process was then implemented to extract key variables, which facilitated the creation of an automatic staging library. This library, combined with a rule-based process, allowed for systematic analysis and evaluation.

**Results:**

Theoretically, the tumor, node, metastasis (TNM) stage was identified as an important prognostic factor for CRC, but it was not selected through feature selection in real-world data. After applying the QMP, rates of missing data were reduced from 75.3% to 35.7% for TNM and from 24.3% to 18.5% for surveillance, epidemiology, and end results across 6491 cases, confirming the system’s effectiveness. Variable importance analysis through feature selection revealed that TNM stage and detailed code variables, which were previously unselected, were included in the improved model.

**Conclusions:**

In sum, we developed a rules-based QMP to address errors and impute missing values in Korea Clinical Data Use Network for Research Excellence data, enhancing data quality. The applicability of the process to real-world datasets highlights its potential for broader use in clinical studies and cancer research.

## Introduction

Medical datasets include various forms of data such as patients’ health status, diagnosis, and treatment information, collected through electronic medical records, diagnostic tests, and treatment records [1]. These data support patient-specific treatment and accurate decision-making by medical professionals [2]. With the growing importance of data-driven medical research, studies using medical data have become increasingly common [3,4]. Advancements in artificial intelligence (AI) and machine learning technologies have further expanded the potential uses of these data, such as for early disease diagnosis and prediction model development [5].

As the volume of medical data grows, infrastructures are being established to analyze and use the data efficiently [6]. Data sharing and linkage enable researchers to access the necessary data more easily. However, challenges such as heterogeneity and incompleteness of datasets remain [7]. For example, during the pseudonymization of integrated medical data, some information may be restricted, and differences in data formats or structures can compromise consistency during adjustment.

Issues such as missing data, inconsistencies, and errors can degrade data quality [8]. Medical data often exhibit imbalance, where some categories of data are underrepresented, which can lead to biased learning and distorted outcomes in AI-based predictive models [9,10]. These quality issues can undermine the reliability of analysis results. Therefore, it is essential to develop a quality management process (QMP) to correct errors and supplement data to improve the quality of medical data and build high-quality datasets. Given the current shortage of specialized personnel trained in handling and managing raw data, it is crucial to manage data quality effectively and enhance usability through systematic and standardized QMPs.

In the medical field, an increasing number of studies have addressed data quality issues [11]. Evaluations of data quality using colon cancer data and proposals for QMPs and frameworks are gaining traction [12,13]. Recently, new methodologies for managing the quality of AI training data have been introduced [14], helping to establish high-quality datasets that meet research purposes for diagnosis and prognosis prediction [15]. While medical data play a decisive role in clinical research and patient treatment, systematic quality management that ensures the consistency, accuracy, and completeness of data is crucial for solving various errors and dealing with missing information [16]. Although comprehensive quality management methodologies for the medical data collection stage are emerging [17], processes applicable to real-world data (RWD) are still lacking.

Therefore, the aim of this study is to develop a QMP for colorectal cancer (CRC) data from the Korea Clinical Data Use Network for Research Excellence (K-CURE). This process was designed to systematically align with the research objectives, identifying key prognostic variables for CRC. We implemented a rule-based approach to improve data completeness and evaluated the effectiveness of the QMP by comparing the data before and after its application.

## Methods

### Stage 1: Planning Stage

#### Data Resources

We used CRC clinical library data established in the K-CURE project at Gachon University Gil Medical Center, approved for use through an institutional review board exemption (GFIRB2024-169). The K-CURE project supports AI-based research and technology development by sharing, providing access to, and linking clinical data from various hospitals. We used a pseudonymized clinical library of 6491 patients with CRC, collected between 2010 and 2022 for the K-CURE project. The pseudonymized clinical library refers to a deidentified dataset in which personally identifiable information has been removed and replaced with pseudonyms. The K-CURE clinical library includes patient information, medical history, diagnoses, cancer staging, test results, treatments, and follow-up data. In addition, structured text-based reports of imaging test results and pathology data from the clinical library were integrated to perform quality management.

#### Ethical Considerations

The study used CRC clinical library data established in the K-CURE project at Gachon University Gil Medical Center, which was approved for use through an institutional review board exemption (GFIRB2024-169). The dataset was pseudonymized, and personally identifiable information was removed and replaced with pseudonyms. Informed consent was waived due to the use of deidentified retrospective data. No compensation was provided to participants. Privacy and confidentiality of patient data were strictly maintained throughout the study.

#### Study Design

In Stage 1, we planned the overall research design to establish a QMP for clinical data that meets our research objectives. To systematize the quality management procedures, we designed a detailed step-by-step process across 4 stages: planning, identification, operation, and evaluation.

**Figure 1.**
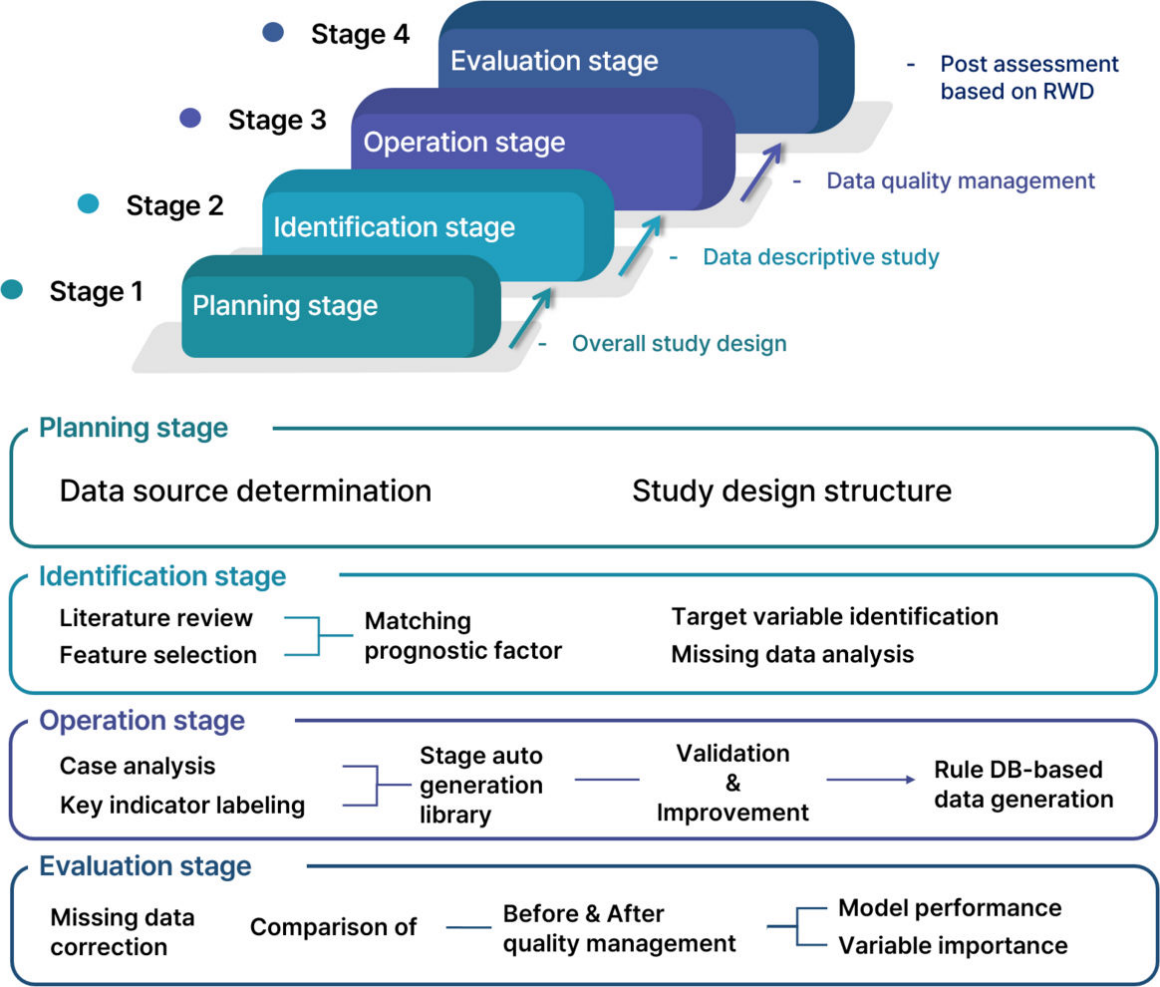
Study design. DB: Database; RWD: real-world data.

In the identification stage, we assessed the general status of the RWD to identify areas requiring quality management. In the operation stage, the QMP was applied to the identified targets. Finally, in the evaluation stage, we compared the pre- and post-quality management results to assess improvements in the data. The overall flow of this study is presented in Figure 1.

### Stage 2: Identification Stage

#### Literature Review to Identify Prognostic Factors

In Stage 2, we conducted a literature review to verify whether the K-CURE CRC data are suitable for constructing a prognostic prediction model. In particular, we sought to identify the key factors influencing the prognosis of patients with CRC and the major variables to consider for constructing a prognostic prediction model for CRC. We searched PubMed for articles published from 2010 to 2024. Our key search terms were (CRC OR colorectal OR CRC) AND (prognosis OR prognostic factor OR predict OR risk factor). The inclusion criteria were as follows: articles published between January 1, 2010, and March 31, 2024, and studies that focused on overall survival, mortality, or 5-year survival as dependent variables. The exclusion criteria included studies with low relevance to the topic or insufficient information on prognostic factors for patients with CRC, and those that discussed only a research design without specific findings. Key influencing factors identified from the selected literature were quantified, and theoretically important factors were derived. These were then used to establish variables for the prognostic prediction model.

#### Feature Selection for Identifying Prognostic Factors

We performed feature selection to identify prognostic factors in the K-CURE CRC data. The Gradient Boosting Classifier was used to evaluate the importance of variables, and the results were compared to theoretically important variables. This model was selected due to its robustness in handling missing values and its effectiveness in evaluating variable importance, which makes it suitable for real-world clinical datasets [18]. Variables with low importance or those inconsistent with the literature review findings were selected as target variables requiring quality management. To conduct quality management, we performed frequency analysis of the major variables of the prognostic prediction model. Then, the error and missing data rates for these target variables were reviewed to examine the overall data distribution. The rate of missing data was calculated using frequency analysis for each variable. Error rates were measured by comparing manually generated stage codes with the data of 164 randomly selected samples, limited to cases without missing data.

### Stage 3: Operation Stage

**Figure 2.**
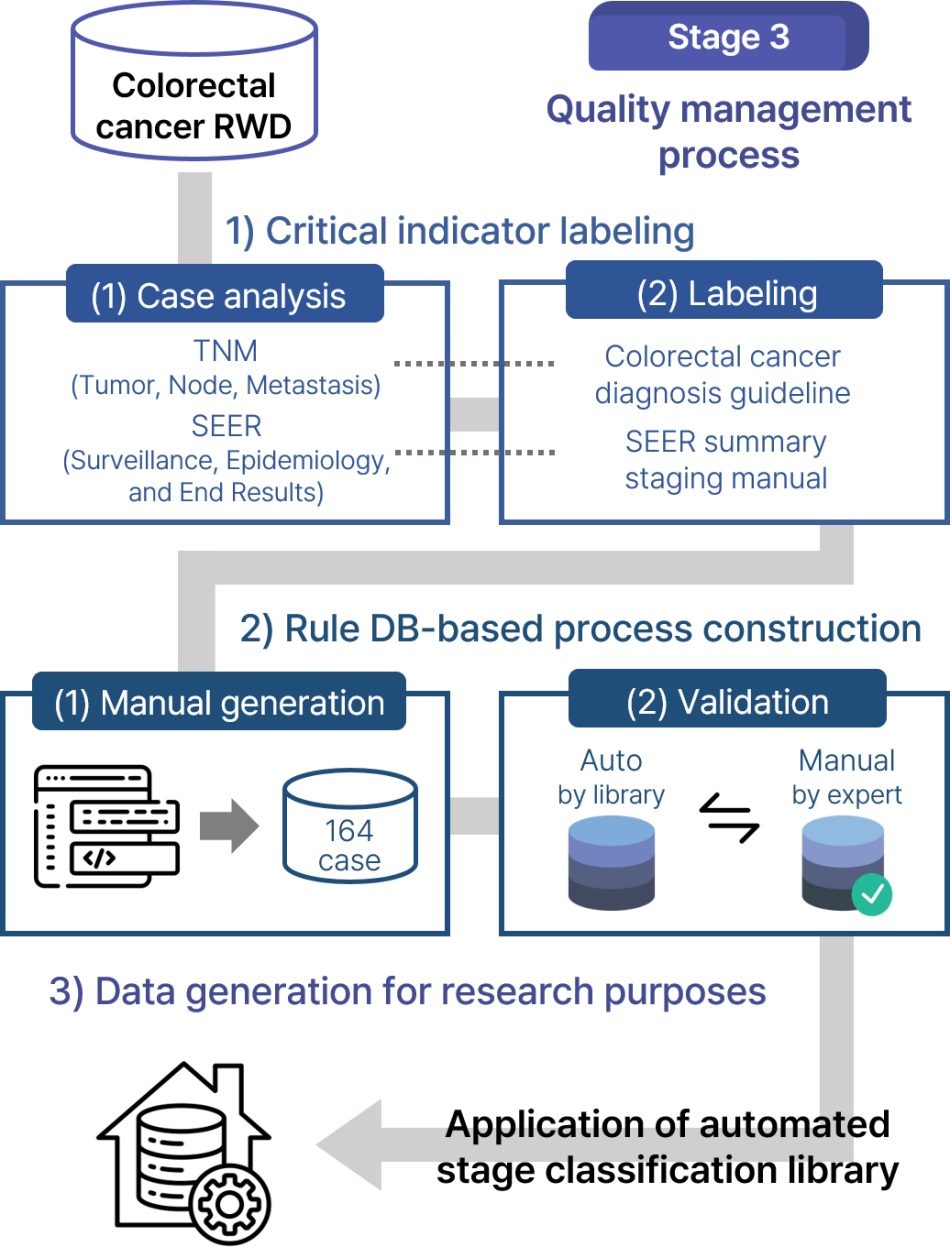
Schematic diagram of our proposed quality management program. RWD: real-world data; SEER: surveillance, epidemiology, and end result; TNM: tumor, node, metastasis.

Figure 2 provides a schematic of the overall QMP.

#### Critical Indicator Labeling for Automated Stage Classification Library

The target variables, tumor, node, and metastasis (TNM) and surveillance, epidemiology, and end results (SEER), are critical indicators for evaluating CRC staging. TNM stage is a standardized cancer stage classification system of the American Joint Committee on Cancer, based on the 8th edition of the American Joint Committee on Cancer Cancer Staging Manual [19]. It evaluates the progression of cancer based on tumor depth, lymph node metastasis, and distant metastasis. SEER summary stage is a standardized cancer staging system widely used in international cancer registration systems to classify how far cancer spreads from the primary site of origin.

Before establishing the QMP, a case analysis was conducted to correct errors and address missing data in the target variables. This analysis involved a detailed review of the TNM and SEER variables of cases in the CRC sample data. We identified cases for which the staging information was omitted or incorrectly recorded to assess the completeness and accuracy of the TNM and SEER variables. We also confirmed whether the missing or erroneous staging information could be supplemented using pathology reports and imaging test results according to a standardized classification system.

To identify key indicators for extracting target variables, we referred to the CRC guidelines, “Korean Clinical Guideline for Colon and Rectal Cancer v.1.0 [20],” and the most recent SEER manual, “Summary Stage 18[21].” Labeling was conducted on specific words and keywords to identify detailed codes for TNM and SEER in the pathology report and imaging test results, respectively. In the labeling process, medical knowledge related to CRC was incorporated to establish coding conditions and patterns for accurate staging extraction.

#### Development of QMPs and Improving CRC Data for Research

In total, 164 cases were randomly selected, and TNM and SEER codes were manually generated for each case. This process adhered to standardized guidelines and protocols for CRC diagnosis and staging classification. To evaluate data quality, the manually generated codes were compared with the corresponding codes in the existing dataset for the same cases, excluding those with missing values. The error rate was calculated based on the number of discrepancies identified through this comparison. The manually generated TNM and SEER code data were also used as reference criteria for validating the automated stage classification library and used as basic data to evaluate the accuracy and consistency of the generated codes.

We evaluated whether the automated library corresponded to guidelines in terms of extracting accurate staging information from clinical data. Then, the accuracy of the library was verified by comparing the concordance between the manually generated TNM and SEER codes and the codes derived from the library. This process focused on the consistency of codes, reasons for discrepancies, and major patterns.

### Stage 4: Evaluation Stage

In Stage 4, the data generated by applying the QMP was evaluated. By comparing the rates of missing data for target variables before and after quality management, we could confirm to what extent the missing values were corrected through the process. Based on the data before and after quality management, initial and improved prognosis prediction models were constructed, and their performances were compared. Model performance was evaluated according to metrics such as accuracy, precision, recall, *F*_1_-score, and area under the receiver operating characteristic curve, to assess whether the application of the QMP improved predictive performance. In addition, we analyzed the impact of target variables on CRC prognosis by checking the importance of variables in the model through feature selection before and after quality management. The prognosis prediction model was constructed using the Gradient Boosting algorithm, and the dependent variable was set as 5-year survival using death information. Python (version 3.12) was used for statistical analysis.

## Results

### Stage 2: Data Descriptive Study Results

Based on the literature review, the most frequently identified prognostic factors were T stage (tumor invasion depth) and N stage (lymph node metastasis), cited in 33 and 32 articles, respectively. Other significant factors included M stage (distant metastasis), the integrated TNM staging system, tumor location, pathological differentiation, and carcinoembryonic antigen levels. Staging may be classified as clinical TNM, pathological TNM, or postneoadjuvant pathological TNM.

As a result of stage 2, variables requiring quality management were identified. A summary of the variables derived from the literature review and feature selection is presented in Table 1. As target variables, we selected TNM stage and SEER, which are theoretically important for prognostic prediction.

**Table 1.** Comparison of literature review and feature selection results.

Factors	Values
Literature review, prognostic factors (n)	
Prognostic factors	N
T stage (depth of invasion)	33
N stage (lymph node metastasis)	32
M stage (distant metastasis)	11
Tumor, node, metastasis staging	18
Tumor grade or pathology	40
Carcinoembryonic antigen (ng/mL)	36
Tumor diameter/length/size (cm)	25
Histological type	20
Neutrophil-to-lymphocyte ratio	15
Adjuvant chemotherapy	20
Liver metastasis	13
Lymphatic invasion	11
Platelet-to-lymphocyte ratio	8
Lymphocyte-to-monocyte ratio	8
Number of retrieved lymph nodes	8
Venous invasion	7
Chemotherapy	7
ECOG (performance status)	7
Vascular invasion	6
Perineural invasion	5
Metastatic site (number of)	5
CA19-9 (U/ml)	5
Glasgow prognostic score	5
American Society of Anesthesiologists grade	5
Feature selection, importance	
Year of initial visit_2022	0.231758
SEER^a^_2.0	0.172401
Age	0.047391
Histological diagnosis_16.0	0.045125
Current_drinking_status_1.0	0.037545
Year of initial visit_2017	0.037528
Perineural invasion_3.0	0.036643
Family history_cancer_1.0	0.033641
Perineural invasion_2.0	0.033559
Perineural invasion_nan	0.033198
Primary site_C18.5	0.02482
Current_smoke_status_nan	0.022937
Histological diagnosis_26.0	0.020962
Histological diagnosis_23.0	0.01356
Primary site_C18.1	0.012204
Lymphatic invasion_2.0	0.011136
TNM_T4N2M1	0.010998
Primary site_C18	0.010715
Primary site_C18.3	0.010592
Primary site_K83.8	0.010291
Molecular_pathology_findings_nan	0.008958
Primary site_C17.0	0.008938
BMI	0.008858

a SEER: surveillance, epidemiology, and end results.

The results of the frequency analysis of the major variables are shown in Table 2. Among the key variables, missing data were observed for height, weight, BMI, total lymph nodes, positive lymph nodes, and the target variables TNM and SEER. The rate of missing data for TNM stage was notably high at 75.3%, while that for SEER was 24.3% across 6491 cases. Moreover, when the error rate was measured using manually generated stage codes from 164 randomly selected samples, the error rate for TNM stage was 50% (43 errors out of 86 nonmissing cases). For the SEER variable, the error rate was 31.1% (47 errors out of 151 nonmissing cases).

**Table 2.** Patient characteristics and missing rates of target variables (N=6491).

Variables and categories	N (%)
Sex, n (%)	
Male	3936 (60.6)
Female	2555 (39.4)
Age, mean (SD)	66.79 (13.4)
Dead, n (%)	
Yes	394 (6.1)
No	6097 (93.9)
5 y survival, n (%)	
Yes	6131 (94.5)
No	360 (5.6)
Height, mean (SD)	162.00 (9.15)
Missing, mean (SD)	2144 (33.0)
Weight, mean (SD)	62.44 (11.88)
Missing, mean (SD)	2135 (32.9)
BMI mean (SD)	23.72 (3.60)
Missing	2146 (33.1)
Total lymph node, mean (SD)	20.25 (12.05)
Missing, n (%)	2633 (40.6)
Positive lymph node, mean (SD)	1.92 (4.40)
Missing, n (%)	2633 (40.6)
Operation, n (%)	
Yes	2631 (40.5)
No	3860 (59.5)
Chemotherapy, n (%)	
Yes	224 (3.5)
No	6267 (96.6)
Radiotherapy, n (%)	
Yes	383 (5.9)
No	6108 (94.1)
Complication after surgery, n (%)	
Yes	524 (8.1)
No	5967 (91.9)
SEER^a^, n (%)	
0	355 (5.5)
1	1818 (28)
2	806 (12.4)
3	192 (3)
4	890 (13.7)
5	14 (0.2)
7	792 (12.2)
9	48 (0.7)
Missing	1576 (24.3)
T stage, n (%)	
0	1 (0)
Tis, n (%)	1 (0)
1	304 (4.7)
2	238 (3.7)
3	814 (12.5)
4	248 (3.8)
Missing	4885 (75.3)
N stage, n (%)	
0	968 (14.9)
1	399 (6.2)
2	235 (3.6)
3	3 (0.1)
4	1 (0)
Missing	4885 (75.3)
M stage, n (%)	
0	1459 (22.5)
1	147 (2.3)
missing	4885 (75.3)

a SEER: surveillance, epidemiology, and end results.

### Stage 3: Data Quality Management

We developed guidelines for creating an automated stage classification library. Examples of critical indicator terms identified for TNM and SEER through labeling are highlighted in italics in Tables3  4, respectively. These guidelines define labeled terms and conditions that allow rule-based automated classification of cancer stage.

**Table 3.** Tumor, node, metastasis stage labeling following the Korean clinical guideline for colorectal cancer v.1.0, with critical indicator terms in italics.

Stage	Labels
Pathology report	
T0	
	No residual tumor
Tis	
	*Confinement to mucosa*
	Invasion to *lamina propria*
	(pTis)
T1	
	Invades *submucosa*
	Invasion to *submucosa*
	Invasion into *submucosa*
	Invasion to *muscularis mucosae*
	(pT1) /(ypT1)
T2	
	Invades *muscularis propria*
	(pT2) /(ypT2)
T3	
	Invades *pericolic adipose tissue*
	Invades *perirectal adipose tissue*
	Invades *subserosa*
	(pT3) /(ypT3)
T4	
	Penetrates *visceral peritoneum*
	Penetration to serosa and perforation
	(pT4a) /(ypT4)
	Direct invades *adjacent organs* or *structures*
	Directly invades adjacent organ
	(pT4b)
N0	
	*No metastasis* in - *regional lymph nodes*
	*No metastasis* in - *pericolic lymph nodes*
	*No metastasis* in - *perirectal lymph nodes*
	*No metastasis* in - *pericolic and perirectal lymph nodes*
	*No metastasis* in - *pericolic and peri-ileal lymph nodes*
	*No metastasis* in - *lymph nodes*
	*No tumor present in* 16 *regional lymph nodes* (0/16)
	(pN0) /(yN0) /(ypN0)
N1	
	Metastasis in *1* of ~ *regional lymph nodes*
	(pN1a) /(ypN1a)
	Metastasis in *2 (or 3)* of ~ *regional lymph nodes*
	(pN1b) /(ypN1b)
	*Tumor deposit* present
	(pN1c) /(ypN1c)
N2	
	Metastasis in *4 (more than)* of ~ *regional lymph nodes*
	(pN2a) /(ypN2a)
	(pN2b) /(ypN2b)
M1	
	*Metastatic adenocarcinoma*
	*Adenocarcinoma, metastatic* from
	*Metastatic colonic adenocarcinoma*
	*Metastatic carcinoma* of rectum
	*Metastatic mixed adenoneuroendocrine carcinoma*
	*Metastatic appendiceal high-grade goblet cell adenocarcinoma*
	*Metastatic mucinous adenocarcinoma*
	*Metastatic mucinous carcinoma*
	*Consistent with metastatic carcinoma*
	*Omental seeding*
Imaging examination results	
T0	
	*No evidence of abnormal wall thickening*
	*No visible definite*
Tis	
	*Tis*
*Invasion of lamina propria*
T1	
	*T1*
*Submucosal invasion*
T2	
*T2*
T3	
*T3*
	*Pericolic (fat) infiltration*
	*Perirectal (fat) infiltration*
	*Mesorectal fat infiltration*
	*Subserosal invasion*
T4	
*T4*
	*T4a /T4b*
	*Visceral peritoneum*
Synonym: LN(s), L/N(s), lymph node(s)^a^	
N0	
*N0*
	*No enlarged*
	*No abnormal enlarging*
	*No pathologic*
	*Nor enlarged*
	*No evidence of regional*
	*No evidence of enlarged*
	*No evidence of enlarged regional*
	*No significant*
	*No significant enlarged*
	*No significant enlargement*
	*No significant enlarged peritumoral*
	*No visible enlarged*
N1	
*N1*
	*Regional*
	*Metastases*
	*Regional metastatic*
	*Regional - metastasis * *(metastases)*
	*Metastatic*
	*With regional lymph node metastasis*
N2	
*N2*
	*Multiple regional metastatic*
	*Multiple regional - * *metastasis/metastases*
	*Several regional - * *metastasis/metastases*
	*Several regional - * *metastasis*
Synonym: metastasis, metastases, metastatic^b^	
M0	
	*No evidence of distant*
	*No evidence of definite distant*
	*No evidence of liver*
	*No evidence of hepatic*
	*No evidence of*
	*Nor distant*
	*Nor or no visible*
	*Rather than*
	*No evidence of enlarged regional L/N or distant metastasis*
M1	
	*Bone*
	*Liver*
	*Hepatic*
	*Pulmonary*
	*Several*
	*No evidence of distant*

a The terms listed as synonyms should be used together with the N stage labels to create the labeling.

b The terms listed as synonyms should be used together with the M stage labels to create the labeling.

**Table 4.** SEER^a^ labeling by Summary Stage 2018, with critical indicator terms in italics.

SEER code	Labels
Pathology report	
0^b^	
	*Intraepithelial*
1^c^	
	Intramucosal
	Confinement in the *lamina propria*
	Invasion to *lamina propria*
	*Confinement to mucosa*
	*Invasion to mucosa*
	*Extension to mucosa*
	*Involvement of mucosa*
	Invasion to *muscularis mucosae*
	*Invades muscularis propria*
	*Invades submucosa*
	*Invasion to submucosa*
	Invasion into submucosa
	Invasion to the submucosa
	*Submucosal invasion*
2^d^	
	Directly invades *adjacent organ*
	Direct invades *adjacent organs or structures*
	Directly invades adjacent organs or structures
	Penetrates *visceral peritoneum*
	Penetration of *visceral peritoneum*
	Invades *subserosa*
	Invades *pericolic adipose tissue*
	Invades *perirectal adipose tissue*
3^e^	
	*Metastasis* in *1* of *regional lymph nodes*
	*With metastasis of pericolorectal lymph node*
	*Tumor deposit*
4^f^	Codes 2+3 (cases corresponding to both Code 2 and Code 3)
7^g^	
	*Metastatic adenocarcinoma*
	*Adenocarcinoma, metastatic from colon or rectum*
	*Metastatic mixed adenoneuroendocrine carcinoma*
	*Metastatic colonic adenocarcinoma*
	*Metastatic carcinoma*
	*Distant lymph node(s)*
9^h^	In cases without evidence
Imaging examination results	
0	—^i^
1	
	*Invasion of lamina propria*
	*Submucosal invasion*
2	
	*Pericolic fat infiltration*
	*Pericolic infiltration*
	*Perirectal infiltration*
	*Perirectal fat infiltration*
3	If the N code is 1 or higher
4	Codes 2+3 (cases corresponding to both Code 2 and Code 3)
7	If the M code is 1 or higher
9	In cases without evidence

a SEER: surveillance, epidemiology, and end results.

b 0: in situ.

c 1: localized only.

d 2: regional by direct extension only.

e 3: regional lymph node(s) involved only.

f 4: regional by both direct extension and regional lymph node(s) involvement.

g 7: distant site(s)/lymph node(s) involved.

h 9: unknown if extension or metastasis.

i Not applicable.

As a result of the evaluation of the automated stage classification library, the concordance rates were 93.3% for TNM and 93.9% for SEER across the 164 cases. By leveraging a rule-based database in the QMP, we were able to supplement missing data in the target variables, resulting in a dataset aligned with the objectives of prognostic prediction.

### Stage 4: Postassessment Based on RWD

**Figure 3.**
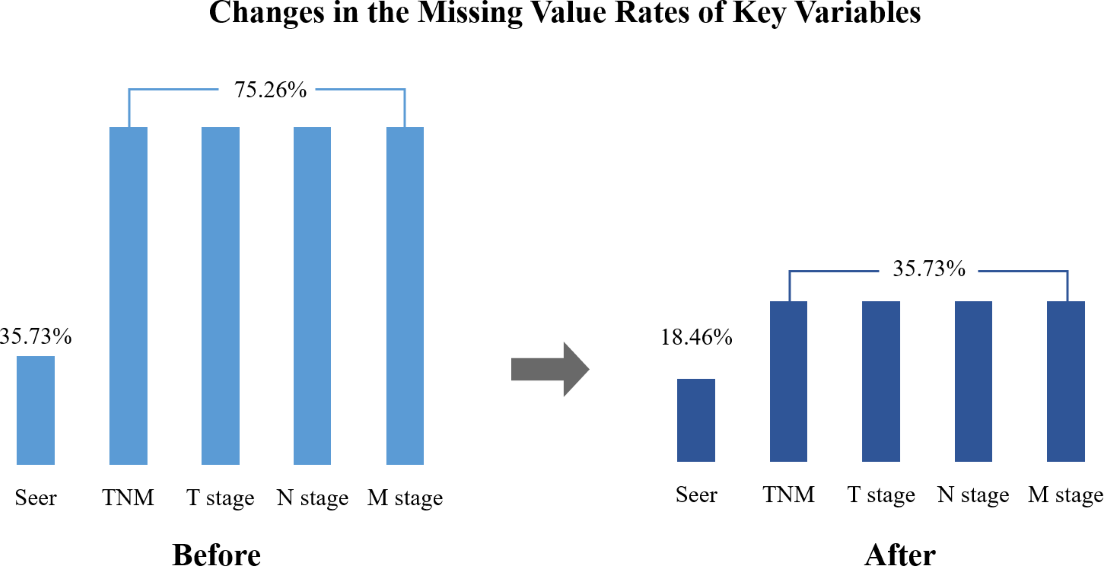
Missing values before and after quality management. SEER: surveillance, epidemiology, and end result; TNM: tumor, node, metastasis.

Comparing the rates of missing data before and after the QMP, the rate decreased from 75.3% to 35.7% for the TNM and from 24.3% to 18.5% for the SEER across 6491 cases. This demonstrates the effectiveness of the QMP (Figure 3).

**Figure 4.**
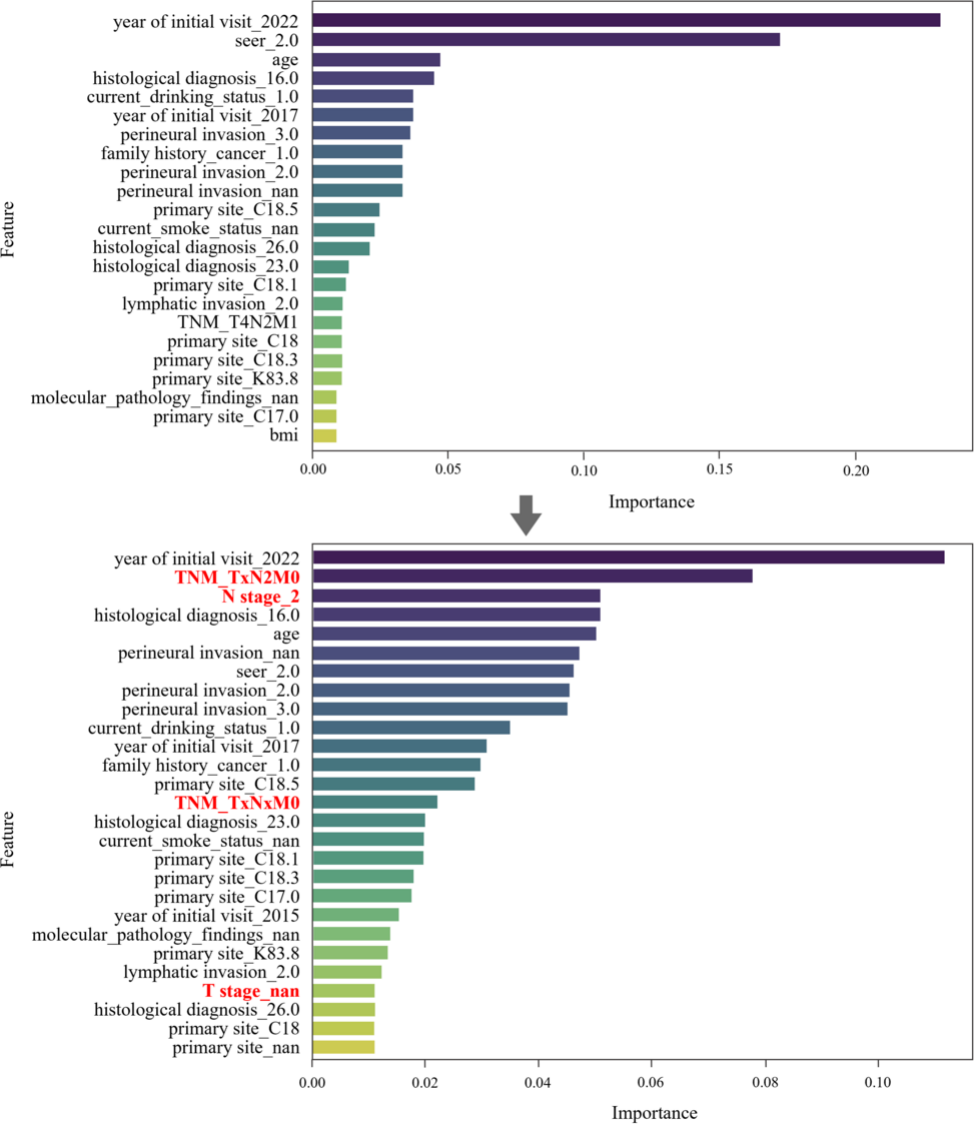
Change in feature importance before and after quality management.

Table 5 presents a comparison of the performance of the models before and after the QMP; a slight improvement was observed. An evaluation of variable importance by feature selection revealed that TNM stage and detailed code variables (T, N, M), which were not identified before quality management, emerged as significant variables after quality management. The variable importance values are shown in Figure 4, and the corresponding importance values are detailed in Table 6. Incorporating these newly identified prognostic indicators into the final model enhances its clinical relevance and interpretability.

**Table 5.** Model performance before and after quality management.

	Before quality management	After quality management
Accuracy	0.933795227	0.9407236336
Precision	0.924949499	0.9279243167
Recall	0.933795227	0.9407236336
*F*_1_-score	0.92898597	0.9330359000
AUROC^a^	0.856226406	0.8724494672

a AUROC: area under the receiver operating characteristic curve.

**Table 6.** Feature importance before and after quality management.

Feature	Importance
Before quality management	
year of initial visit_2022	0.23176
SEER_2.0	0.17240
Age	0.04739
histological diagnosis_16.0	0.04513
current_drinking_status_1.0	0.03755
year of initial visit_2017	0.03753
perineural invasion_3.0	0.03664
family history_cancer_1.0	0.03364
perineural invasion_2.0	0.03356
perineural invasion_nan	0.03320
primary site_C18.5	0.02482
current_smoke_status_nan	0.02294
histological diagnosis_26.0	0.02096
histological diagnosis_23.0	0.01356
primary site_C18.1	0.01220
lymphatic invasion_2.0	0.01114
TNM_T4N2M1	0.01100
primary site_C18	0.01072
primary site_C18.3	0.01059
primary site_K83.8	0.01029
molecular_pathology_findings_nan	0.00896
primary site_C170.	0.00894
BMI^a^	0.00886
After quality management	
year of initial visit_2022	0.11148
TNM^b^_TxN2M0	0.07741
N stage_2	0.05068
histological diagnosis_16.0	0.05061
age	0.05013
perineural invasion_nan	0.04725
SEER^c^_2.0	0.04599
perineural invasion_2.0	0.04532
perineural invasion_3.0	0.04489
current_drinking_status_1.0	0.03479
year of initial visit_2017	0.03067
family history_cancer_1.0	0.02972
primary site_C18.5	0.02883
TNM_TxNxM0	0.02201
histological diagnosis_23.0	0.01986
current_smoke_status_nan	0.01954
primary site_C18.1	0.01947
primary site_C18.3	0.01790
primary site_C170.	0.01747
year of initial visit_2015	0.01526
molecular_pathology_findings_nan	0.01374
primary site_K83.8	0.01342
lymphatic invasion_2.0	0.01218
T stage_nan	0.01105
histological diagnosis_26.0	0.01104
primary site_C18	0.01093
primary site_nan	0.01088

a BMI: body mass index.

b TNM: tumor, node, metastasis.

c SEER: surveillance, epidemiology, and end result

## Discussion

### Principal Findings

This study proposed a QMP to generate high-quality data. We used the K-CURE dataset to develop the QMP and applied it to a CRC clinical library to evaluate the quality improvement effects. After applying the process, TNM stage and individual T, N, and M codes emerged as important factors when constructing a prognostic model. This suggests that the proposed QMP can create high-quality data for research.

Gaps in datasets can occur due to direct omissions of data, limitations in data collection, and technical issues [22,23]. Missing values may arise due to patient movement, treatment interruptions, or omitted tests or procedures, resulting in the loss of important variables. Various methods, such as statistical imputation or ML-based techniques, have been proposed to address missing data but often fail to fully reflect the complexity of clinical environments [24,25]. This reduces the reliability of data over the long term, affecting dataset quality and reducing the reliability of findings.

Various basic statistical methods, such as imputation, have been used to address missing data [26-28]. More recently, ML-based methods such as K-nearest neighbor [29], matrix factorization [30], and random forest approaches have also emerged [31]. These methods are effective when missing data are not random and do not follow specific patterns, as they learn from the dataset itself and predict missing values [32]. This makes them relatively insensitive to the rates or patterns of missing data. Novel techniques such as attention-based models [33] or the large language model forest framework have also been applied [34]. However, previous studies have focused on evaluating and replacing missing values, rather than applying multistage processes to improve overall data quality.

In this study, we reviewed several previous studies on CRC to construct an improved dataset and identify prognostic factors. For clinical research, it is crucial to identify and evaluate factors with strong evidence-based associations with prognoses [35]. However, in our study, theoretically important variables were not always selected from the actual data, and some missing values could not be addressed through the QMP. This indicates that there was a lack of information on important variables during the initial stages of data construction. Therefore, important prognostic variables should be thoroughly reviewed and systematically managed from the initial stages of data construction.

Using CRC staging guidelines, we performed labeling by extracting text-based terms from pathology reports and imaging test results to establish a rule-based QMP. Recently, there has been a trend toward research focusing on developing rule-based quality management and quality assessment methodologies using medical data. This expands the possibility of systematically detecting and correcting errors in data [36]. This approach effectively analyzes clinical quality issues, improves data accuracy, and provides reliable information for clinical research and decision-making [37]. Such a strategy has been found to be applicable to real-world medical data [38]. The QMP developed in this study shows the utility of rule-based systems, generating data with improved completeness. Applying this approach could provide accurate data for future prognostic prediction and decision support systems.

Traditional quality management methodologies focus on preventing and correcting errors during data construction and operation [39]. For example, such methods often rely on automated systems or checklists to minimize input errors or to validate the accuracy of collected data [40]. However, we propose a rule-based QMP that identifies and corrects missing values and errors in datasets that are already established. This approach not only addresses potential issues that can occur during the data construction phase, but also facilitates the detection and resolution of missing data that arise during data analysis.

Recently, there have been active attempts in medical research to develop QMP systems using various clinical and public datasets, including electronic medical record data [41-43]. This approach is essential for institutions with large-scale medical datasets and platforms built from multiple integrated datasets. In multi-center research, a method to prioritize data quality dimensions and key evaluation variables, supported by feedback systems to monitor and assess data quality, has been proposed. This study provides a foundation for the automation of future QMP systems and the development of new approaches using AI and ML, enhancing the usage of medical data by researchers in public data platforms.

We focused on addressing missing data for quality management; we have not proposed a comprehensive solution for various data errors in clinical environments. Also, a limitation is the complexity of clinical staging decisions—involving multidisciplinary discussions, treatments such as neoadjuvant therapy, and surgical findings—which can lead to discrepancies or missing values in retrospective research data. This complexity may influence the interpretation of the study results and may affect the generalizability of the data. Nonetheless, this work is important in that we propose a systematic process to improve the quality and applicability of real-world medical data. Future efforts should consider advanced processes that address the entire data lifecycle, from construction to usage and operation.

### Conclusion

We developed a rule-based QMP that improves data quality and identifies key prognostic factors in CRC datasets. Although missing data and other complex challenges in real-world clinical data remain, the approach demonstrates the utility of systematic quality management. Future work should expand the QMP to address diverse data errors across the data lifecycle.

## Abbreviations

AI: artificial intelligence
CRC: colorectal cancer
K-CURE: Korea Clinical Data Use Network for Research Excellence
QMP: quality management process
RWD: real-world data
SEER: Surveillance, Epidemiology, and End Results
TNM: tumor, node, metastasis
